# Supplementary material for: Phenotypes of Floral Nectaries in Developmental Mutants of Legumes and What They May Tell about Genetic Control of Nectary Formation
Source: Biology (Basel). 2022 Oct 19;11(10):1530. doi: 10.3390/biology11101530 (PMC9598078; doi:10.3390/biology11101530)
Supplement: Supplementary file 1 [file biology-11-01530-s001.zip › Figure_S1.pdf]

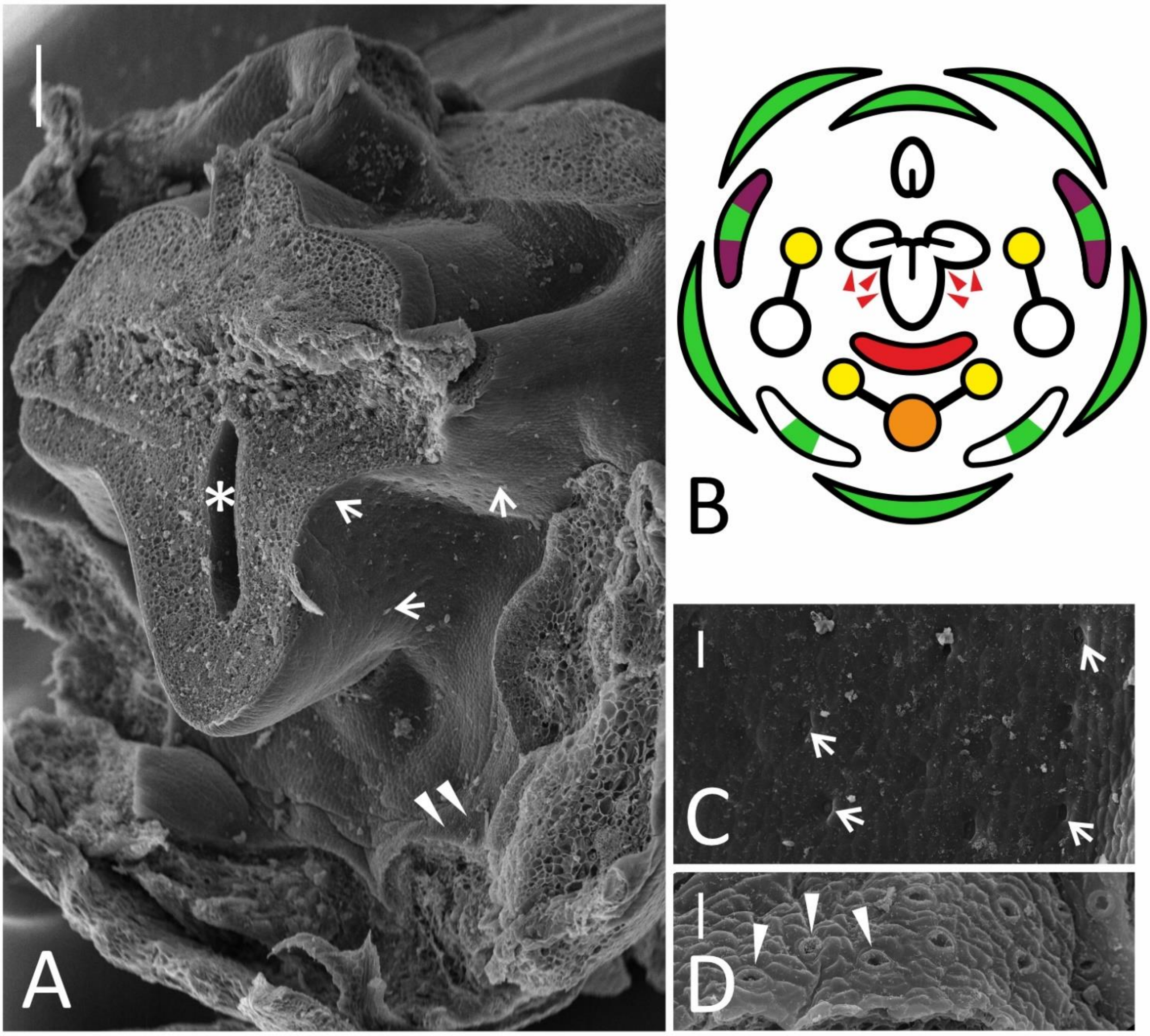

Figure S1. Morphology of late flowers of pea line JI2163 (*stp-1*) with severe anomalies represented as SEM image (**A**) and floral diagram (**B**). Both (**A**) and (**B**) are oriented with their abaxial sides downwards. Stomata-bearing areas in the abaxial part of receptacle and on carpels' bases are enlarged on (**C**) and (**D**), respectively. Key: arrows = exemplary stomata on carpels' bases; white arrowheads = exemplary stomata on receptacle; asterisk = base of the central carpel; red arrowheads = area where stomata are found on carpels' bases. For color designations on floral diagram, see Figure 1 in the main text. Scale bars: 300 μm (**A**), 30 μm (**C**, **D**).
