# Supplementary material for: Phenotypes of Floral Nectaries in Developmental Mutants of Legumes and What They May Tell about Genetic Control of Nectary Formation
Source: Biology (Basel). 2022 Oct 19;11(10):1530. doi: 10.3390/biology11101530 (PMC9598078; doi:10.3390/biology11101530)
Supplement: Supplementary file 1 [file biology-11-01530-s001.zip › Figure_S2.pdf]

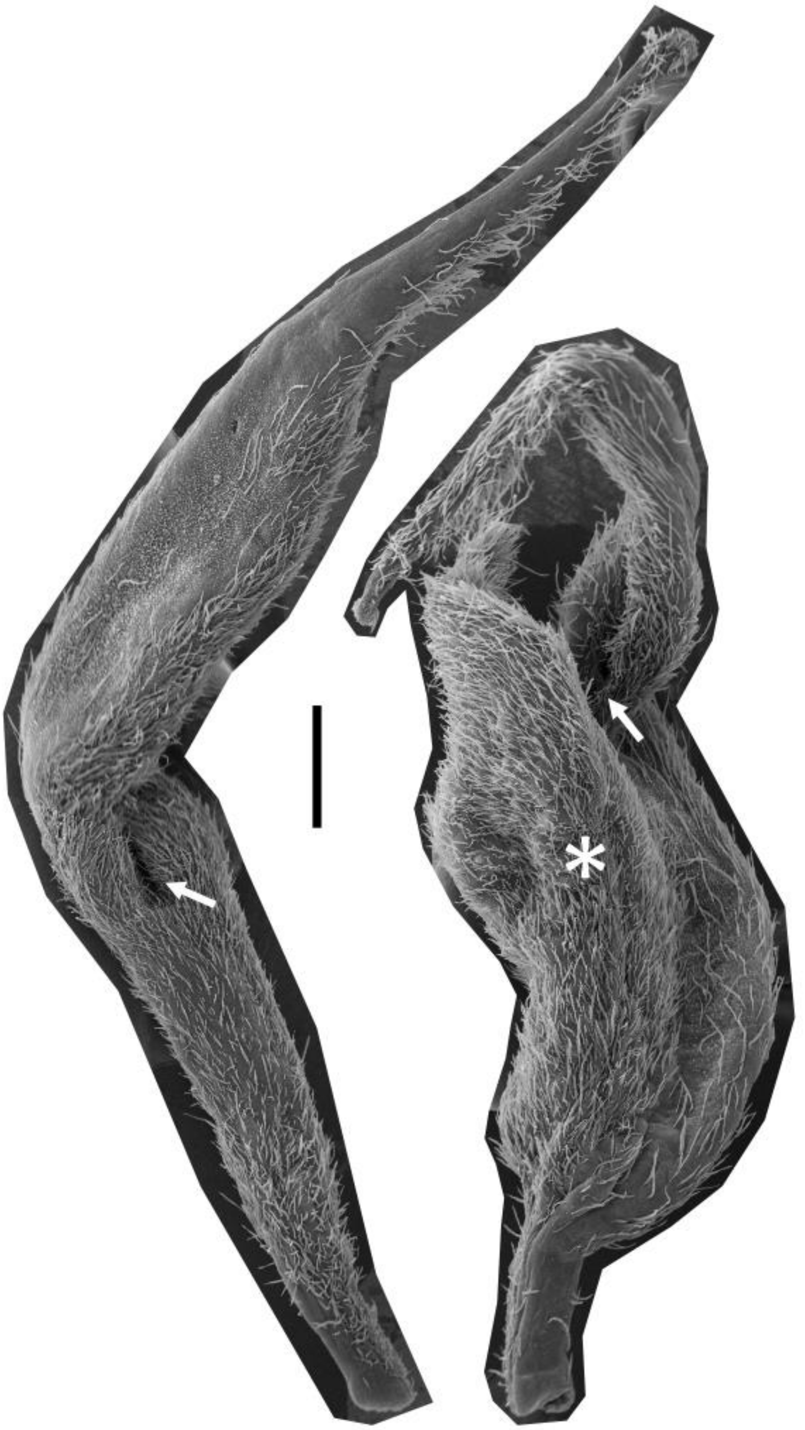

Figure S2. Carpel morphology in *Wisteria floribunda* f. *violaceoplena*. Key: arrow = imperfect carpel closure; asterisk = carpel-like appendage. Scale bar: 1 mm.
