# Supplementary material for: Phenotypes of Floral Nectaries in Developmental Mutants of Legumes and What They May Tell about Genetic Control of Nectary Formation
Source: Biology (Basel). 2022 Oct 19;11(10):1530. doi: 10.3390/biology11101530 (PMC9598078; doi:10.3390/biology11101530)
Supplement: Supplementary file 1 [file biology-11-01530-s001.zip › Figure_S3.pdf]

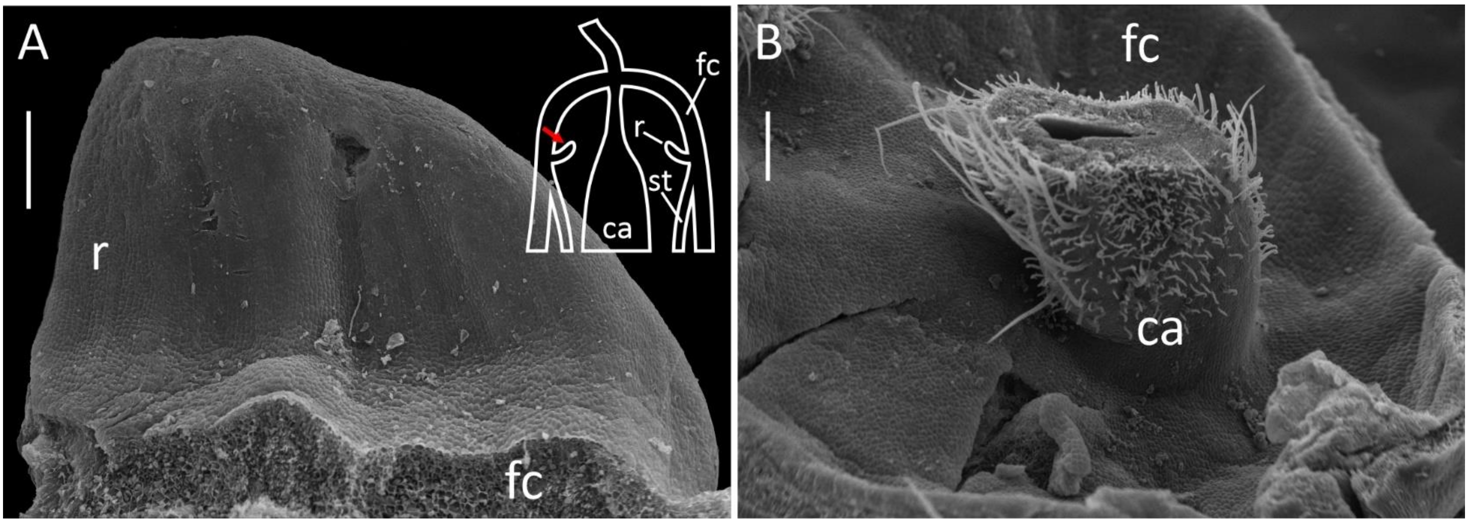


**Figure S3.** The inner surface of an annular ridge (**A**) and floral cup (**B**) of wild-type flowers of *Clitoria ternatea*. Inset (**A**) is a schematic representation of floral structure of this species. Key: red arrow = place illustrated in (**A**); ca = carpel base; fc = floral cup; r = ridge; st = stamens. Scale bars: 300 μm.
