# Supplementary material for: Phenotypes of Floral Nectaries in Developmental Mutants of Legumes and What They May Tell about Genetic Control of Nectary Formation
Source: Biology (Basel). 2022 Oct 19;11(10):1530. doi: 10.3390/biology11101530 (PMC9598078; doi:10.3390/biology11101530)
Supplement: Supplementary file 1 [file biology-11-01530-s001.zip › Figure_S4.pdf]

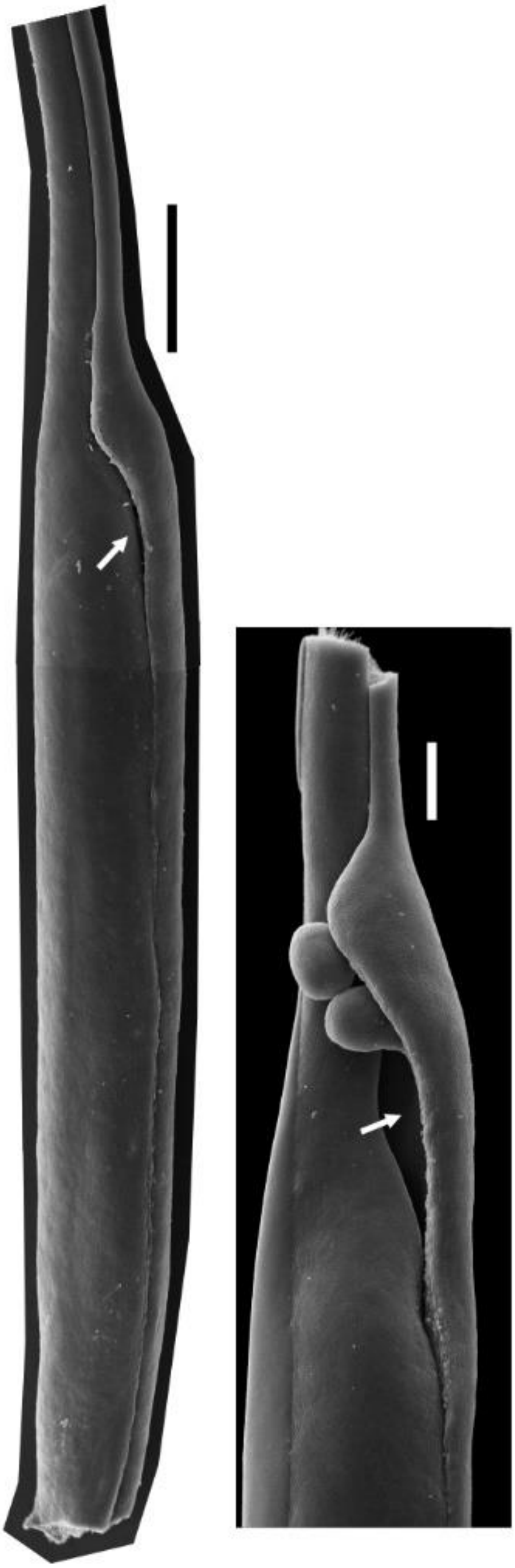

Figure S4. Carpel morphology in *Caragana arborescens* f. *lorbergii*. Note unprotected ovules on right photo. Key: arrow = imperfect carpel closure. Scale bars: 1 mm (left), 300 μm (right).
